# Stan Scheller: The Forerunner of Clinical Studies on Using Propolis for Poor and Chronic Nonhealing Wounds

**DOI:** 10.1155/2013/456859

**Published:** 2013-04-24

**Authors:** M. Kucharzewski, S. Kubacka, T. Urbanek, K. Wilemska-Kucharzewska, T. Morawiec

**Affiliations:** ^1^Department of Descriptive and Topographic Anatomy, Medical University of Silesia, Ulica Jordana 19, 41-808 Zabrze, Poland; ^2^Outpatient Surgery Center No. 2, Specialist Hospital No. 2, Ulica Batorego 15, 41-902 Bytom, Poland; ^3^WZOZ Mother and Children, Ulica Sobieskiego 7A, 42-200 Częstochowa, Poland; ^4^Department of General and Vascular Surgery, Medical University of Silesia, Ulica Ziołowa 45/47, 40-635 Katowice, Poland; ^5^Department of Oral Surgery, Faculty of Medicine and Dentistry, Medical University of Silesia, Plac Akademicki 7, 41-902 Bytom, Poland

## Abstract

For hundreds of years poor and chronic nonhealing wounds have constituted a serious problem to medicine. What is more, treating such wounds is an expensive let alone a long-lasting process. The following paper describes Professor Scheller's achievements in using propolis for poor and chronic non-healing wounds. The authors' intention was to present the results connected with the use of the ethanolic extract propolis, in the treatment of patients suffering from burns, venous crural ulceration, local sacral bone pressure ulcers, suppurative osteitis and arthritis, suppurative postoperative local wound complications, and infected traumatic wounds.

Needless to say, Professor Stan Scheller was born on January 7, 1928 in Lviv. When he was a student, in 1950 he commenced a job as the junior assistant under the leadership of Professor Ludwik Hirszfeld, in the Department of Microbiology of the Medical Academy in Wroclaw (1950–1953). Then in the Microbiology and Immunology of the Silesian Medical Academy in Zabrze, Rokitnica, he worked first as an assistant (1960–1962), later as a tutor (1962–1971), assistant professor (1971–1985), associate professor (1985–1997), and finally professor (1997-1998). From October 1, 1976 he become the Head of the Microbiology Department and continued his scientific and academic work until he retired on the 1st of October 1998.

Taking into account Scheller's achievements in the clinical implementation of propolis, the authors retrospectively review as well as present Scheller's experience concerning an introduction and an implementation of propolis in the chronic wound treatment.

In 1966 Stan Scheller decided to undertake research concerning propolis. He discovered and described his own method of the preparation of the ethanol extract of propolis (EEP). 

It would be worth noting that propolis was collected manually, in beehives located in the south of Poland (The Carpathians, Nowy Sącz region). Propolis was kept desiccated pending its processing. It was extracted in 95% (v/v) from ethyl alcohol, in a hermetically closed glass vessel for 4 days at 37°C, and also underwent occasional shaking. The ethanolic extract was then filtered through a Whatman filter paper no. 4 and evaporated in rotary evaporator, under reduced pressure at 60°C [1–3]. 

The following largely enabled the examinations of its biological properties. To exemplify, he managed to indicate the presence of 19 particles in the propolis containing solution as well as proved their antiseptic effect on the Gram-positive bacteria [4]. It would be worth noting that the name of the extract has been universally acknowledged in the worldwide scientific literature. Professor Scheller was also the first to use ethanol extract of propolis for poor and chronic nonhealing wounds [1, 5]. 

Chronic wounds, including pressure ulcers, chronic leg venous ulcers, diabetic foot ulcers, burns, and other kinds of wound healing by secondary intention, are common in both acute and community setting. The treatment of these wounds comprises a number of strategies including debridement, the use of various wound dressing, antimicrobial agents, footwears, and physical therapies, that is, compression stocking or other local treatment methods [6].

What would be also worth mentioning is the fact that propolis is a resinous substance collected from trees by the bee *Apis mellifera*, which uses it as a building and insulating material in the hive. Furthermore, bees make use of propolis (bee glue) not only as a building material but also as a material to keep a low concentration of bacteria and fungi in the hive. Although its chemical composition varies, propolis generally includes about 10% of essential oils, 5% of pollen, and 15% of various organic polyphenolic compounds including flavonoids and phenolic acids. Propolis is remarkably used in dermatology for wounds healing, burn and topical ulcers treatment, healing time reduction, wound contraction increase, and tissue repair acceleration. During the wound-healing processes synchronized cellular and molecular interactions take place to repair the damaged tissue [7–9].

The following beneficial aspects connected with propolis are therapeutic properties which can positively influence the wound-healing processes. Moreover, the undertaken in vitro antibacterial activity was verified against several Gram-positive and Gram-negative bacteria which is said to result from synergism between propolis compounds, mainly pinocembrin and galangin flavonoids [8–12]. 

Furthermore, one of the first analyses concerning clinical efficacy of propolis which was prepared and applied in accordance with the Scheller's method was described in 1975 [1], whereas the clinical material description concerned the cases of the poor and chronic non-healing wounds, in 100 patients treated between 1972 and 1974, in the Department of Dermatology in Hospital No. 2 in Bytom, the Clinic of General Surgery of the Silesian Medical Academy in Bytom, and the Clinic of Orthopedic Surgery of the Silesian Medical Academy in Bytom. The research which concerned the above mentioned group of was conducted by Dr. Kubacka under the leadership of professor Scheller [1]. The target group, who underwent clinical assessment, consisted of 12 patients with burns, 30 with venous crural ulceration, 10 with local sacral bone pressure ulcers, 23 with suppurative osteitis and arthritis, 15 with suppurative postoperative local wound complications, and 10 with infected traumatic wounds. All the 100 patients, described in the research presented in Dr. Kubacka Ph.D. thesis, had been unsuccessfully treated in the previous dermatological, orthopedic, and/or surgical hospitals. The durations of the disease, according to the wound etiology, were shown in Table 1 [1]. What follows is that according to Scheller's elaborated method and experience, all the patients were treated with 3% ethanol propolis solution, prepared by the Department of Microbiology, Silesian Medical Academy in Zabrze, Rokitnica. Moreover, the patients with osteitis and fistulas underwent wound rinsing every day, whereas on the rest of the patients the EEP dressings were applied, which were changed every day.

The patients with the II burn grade were treated with EEP preparations for approximately 10 days and those with grade III burns for up to 6 weeks. In each case the wounds were reported to be healed. It would be worth noting that an interesting case presented by Kubacka and Scheller. It concerned a 9-month-old baby who suffered from electric burns on eye and cheek, and the above resulted in a massive and deep skin defects which responded well to EEP treatment and managed to heal properly (Figures 1 and 2). Another case involved a 4-year-old girl with burns on her fingers (Figures 3 and 4).

In the group of 30 patients with venous crural ulceration, the wounds were healed in 16 cases and alleviated in 11 cases. The wounds failed to show healing processes in 3 cases who suffered from lymphatic leukemia. The examples of the ulcerations treated by Scheller are presented in Figures 5 and 6.

Among patients with pressure ulcers in sacral bone region, 9 wounds were reported to be healed. Only two patients did not respond to the treatment. Some of the examples were presented in Figures 7 and 8. 

The chronic osteomyelitis and arthritis in lower limbs were accompanied by multiple fistulas, in one case, and even 20 fistulas were recorded. What would be worth noting is that 8 of the patients had been unsuccessfully treated for a few months, in the period of 5 years. Yet 15 of them were successfully cured after the application of the EEP therapy. The examples are presented in Figures 9 and 10. 

The infectively treated postoperative wounds, underwent a following treatment the duration of which ranged from 2 weeks up to 2 months. Upon the application of EEP dressings the unpleasant odor, edema, and flare around the wound were largely reduced. The bacteriological examination further revealed the lack of bacteria in the wound exudate. It would be of worth to note that ten wounds were reported to heal completely while the rest (5 of 15) indicated no exudates at all. All in all, the described material, which included 100 patients treated in accordance with the method put forward by Scheller, revealed that 66% of the wounds were thoroughly healed, 21% were significantly alleviated, and only in the 13% of cases the failure was noticed, mainly in the group of patients with osteomyelitis, crural venous ulcer, or pressure ulcer [1].

 As previously mentioned and clinically confirmed, propolis is said to have several therapeutic properties, such as antibacterial, anti-inflammatory, healing, anesthetic, anticarcinogenic, antifungal, antiprotozoan, and antiviral activities [10–17]. Added to that, propolis contains copper 26.5 mg/kg, manganese 40 mg/kg, and the ash residue contains iron, calcium, aluminum, vanadium, strontium, and silicon, vitamins such as B1, B2, B6, C, and E, and a number of fatty acids [18]. In addition, it also includes some enzymes such as succinic dehydrogenase, glucose-6-phosphatase, adenosine triphosphatase and acid phosphatase [18]. What would be worth mentioning is that when Professor Scheller conducted his pioneering research, he was largely unfamiliar with the above mentioned properties of propolis [19–21]. 

Furthermore, Professor Scheller and others published a number of works dealing with the biological properties as well as the clinical application of propolis on cartilaginous tissue, the regeneration of bone tissue, and on dental pulp regeneration [5, 22, 23]. 

Apart from that, he successfully introduced the use of the EEP in poor healing wound treatment. What followed was that the subsequent years resulted in a number of publications which confirmed his research, let alone presented the influence of the application of propolis on wounds. 

Moreover, Khayyal et al. [24] reported that aqueous propolis extract possesses significant anti-inflammatory properties and has successfully reduced oedema in both acute and chronic models of inflammation. Similar to the Kubacka and Scheller group of patients the application of high efficiency of the propolis solution in patients with chronic osteomyelitis was also observed, probably due to its antioxidant, anti-inflammatory and antimicrobial effects [1].

Propolis has also been used in the treatment of cutaneous lesions such as burns, wounds, and ulcers by other authors. In addition to the above, Morels and Garbarino [25] used a hypoallergenic formula of propolis and obtained a very satisfactory evolution and cicatrisation in cases of wounds with and without infection. A fast improvement, shorter healing time, let alone low number of septic complications were also obtained. The cicatrisation was evident between the 4th and 5th day by the early formation of granulation tissue. The antimicrobial capacity was also noticed clinically with the fast regression of the infective compound of the suppurated wound.

In the study published by Kurson [26], sixty-four patients with tibial skin ulcers, aged from 23 to 98 years old, were treated using propolis extract in an ointment form. The ointment was applied daily to the ulcerated area, which was also treated peripherally with antibiotic ointments. The treatment lasted for 4–12 weeks. At the end of the treatment, 19 out of the 84 treated patients displayed no clinical signs of the active ulceration; in further 19 cases the clinical improvement of the local condition was observed [26]. 

Propolis was also utilized in another trial of hospital patients with infected wounds. Propolis improved wound-healing rates and reduced local infection severity. Over half of infective bacteria were eliminated within 4 days. Propolis did not produce antibiotic resistance strains of the bacteria [27]. 

The review of the available literature clearly envisages that each year more and more papers concerning the influence of propolis on wound-healing process are published. The mechanisms of the propolis activity have been studied on the clinical as well as molecular level. Consequently, it can be clearly stated that Professor Stan Scheller's studies regarding the influence of propolis on the poor healing and chronic non-healing wound proved to be of great importance to science.

## Figures and Tables

**Figure 1:**
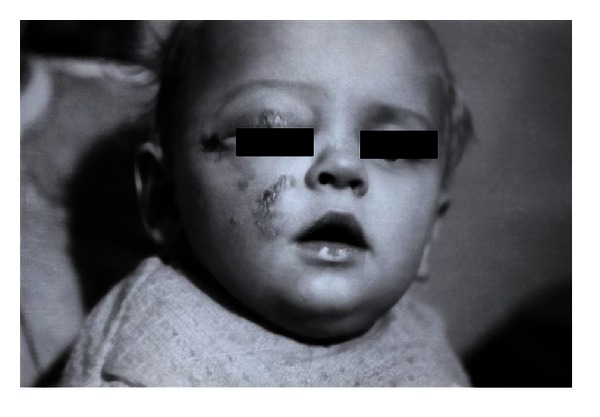
A 9-month-old baby showing deep electric burns in its eye and cheek regions (reproduction of the picture with Dr. Kubacka permission).

**Figure 2:**
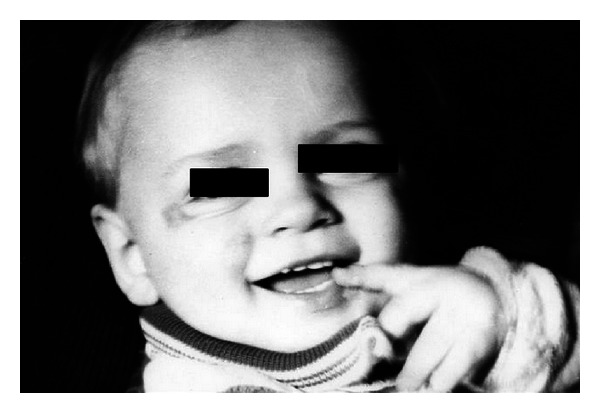
The same baby after EEP treatment (reproduction of the picture with permission).

**Figure 3:**
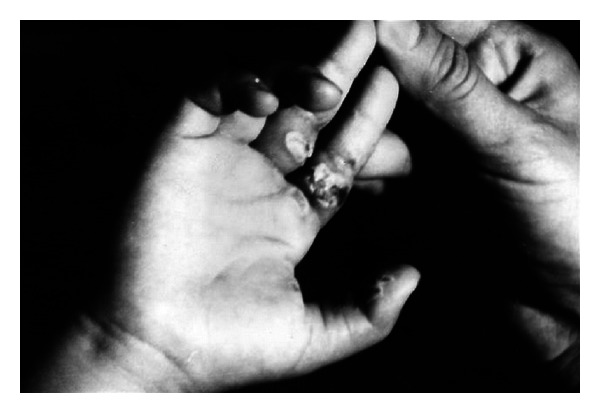
A 4-year-old girl with burns on her fingers (reproduction with permission).

**Figure 4:**
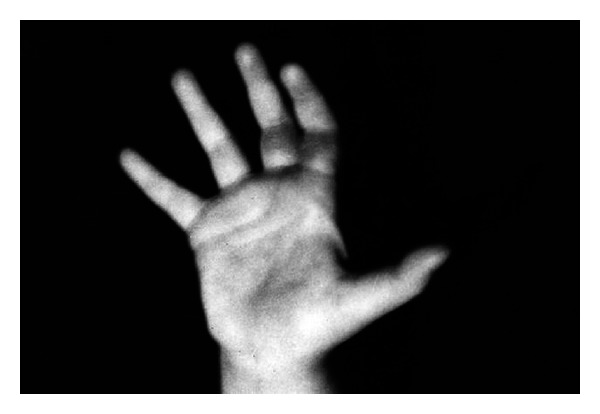
The same girl after EEP treatment (reproduction with permission).

**Figure 5:**
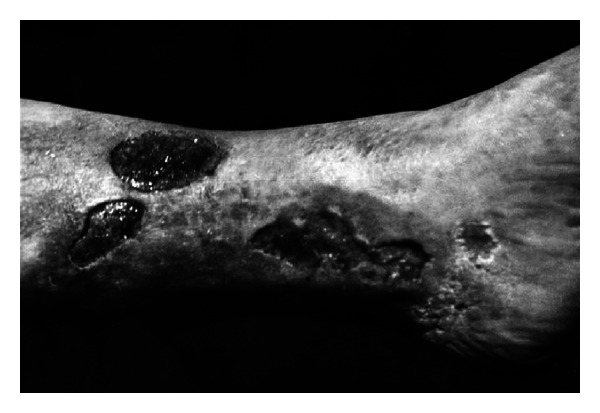
A 59-year-old woman showing venous crural ulceration, under treatment for 10 years (reproduction with permission).

**Figure 6:**
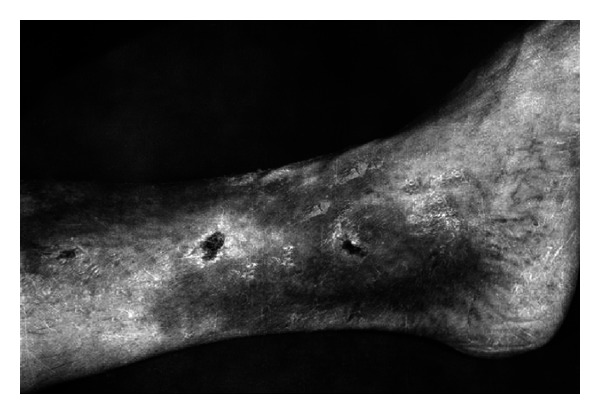
The same woman after EEP treatment (reproduction with permission).

**Figure 7:**
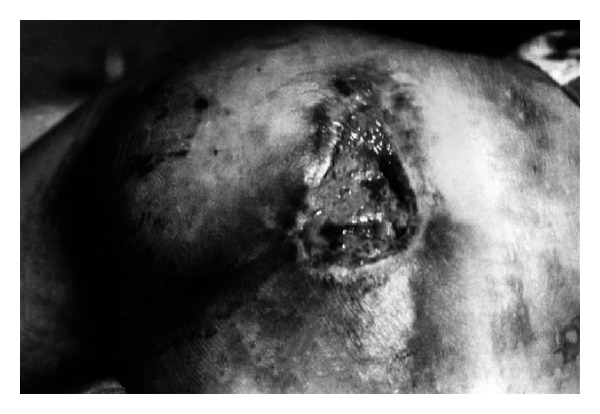
A man showing pressure ulcers in sacral bone region (reproduction with permission).

**Figure 8:**
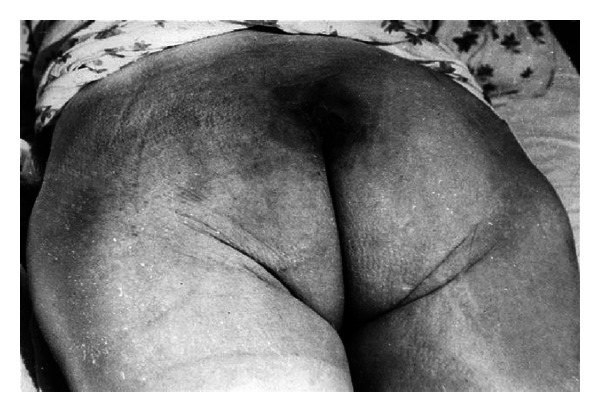
The same man after EEP treatment (reproduction with permission).

**Figure 9:**
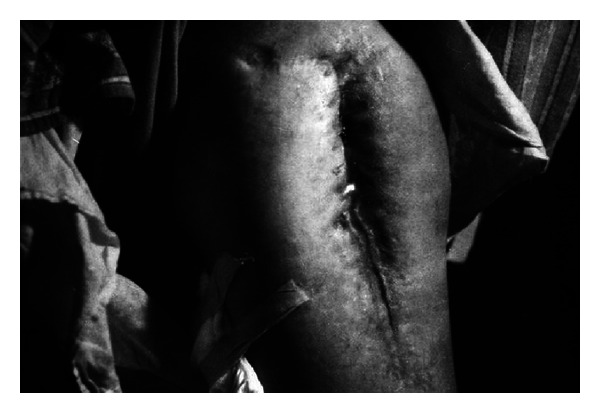
A 42-year-old man with multiple suppurative fistulas in his hip joint, under treatment for 4 years (reproduction with permission).

**Figure 10:**
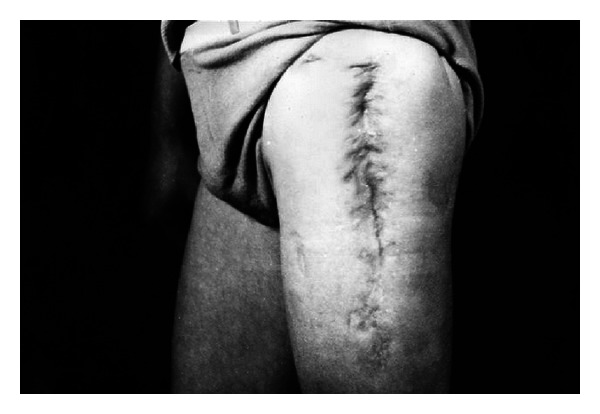
The same man after EEP treatment (reproduction with permission).

**Table 1:** The characteristics of the group and the results of the wound treatment in Kubacka and Scheller patient cohort.

	Number of patients	Duration of illness	Wound healing	Significant improvement	Failure
Burns	12	4 hours to 2 weeks	11	1	
Crural ulceration	30	3 months to 30 years	16	11	3
Pressure ulcers	10	4 months to 5 years	5	3	2
Osteomyelitis	23	2 years to 5 years	15		8
Infection of the wound after trauma/injury	10	2 days to 7 days	9	1	
Infection of the operative wound	15	1 week to 6 months	10	5	
